# Internal limiting membrane detachment in acute Central Retinal Artery Occlusion: clinical features, multimodal imaging, outcomes & prognostic biomarker

**DOI:** 10.1186/s40942-022-00436-7

**Published:** 2022-12-28

**Authors:** Mukesh Jain, Raja Narayanan, Biswajit Barik, Niroj Kumar Sahoo, Vishal Raval, Nikitha G. Reddy

**Affiliations:** Anant Bajaj Retina Institute, L.V. Prasad Eye Institute, Hyderabad, Telangana India

**Keywords:** Central retinal artery occlusion, Internal limiting membrane detachment, Optical coherence tomography, Biomarker

## Abstract

**Purpose:**

To report the clinical features, multi-modal imaging characteristics and their corroboration, and prognostic value of internal limiting membrane detachment (ILMD), a novel OCT biomarker in acute CRAO.

**Design:**

Retrospective observational case-control study at institutional tertiary eye care centers.

**Methods:**

60 eyes of 60 patients of acute CRAO with optical coherence tomography (OCT) at baseline were included. Eyes were grouped in (a) With ILMD; (b) With no-ILMD. Multimodal imaging correlation, BCVA change and binary logistic regression were studied.

**Results:**

Eighteen eyes (30%) were noted to have ILMD. At presentation, ILMD on OCT corroborated with macular non-perfusion with enlarged foveal avascular zone both on OCT-angiography (OCTA) and fundus fluorescein angiography (FFA). On follow-up, ILMD had resolved in all cases with fragmentation, disruption and atrophy of the retinal layers. Logistic regression showed poor baseline visual acuity was significantly associated with the odds of ILMD [Odds Ratio (OR) 31.02, p = 0.0018, 95% confidence interval: 1.81–529] while controlling for potential confounders including age (p = 0.60), gender (p = 0.316) duration of symptoms (p = 0.114), follow-up duration (p = 0.450) and final BCVA (p = 0.357). Eyes with ILMD and no-ILMD had a baseline BCVA of 2.62 LogMAR (light perception) and 2.05 LogMAR (Snellen equivalent 20/2000), respectively. On follow up, none of the eyes with ILMD showed any improvement. In contrast, nine (21.4%) eyes in no-ILMD had a vision of 20/400 and above with a mean final visual acuity of 1.87 + 0.78 LogMAR (p = 0.000).

**Conclusion:**

ILMD correlated with macular non-perfusion and poor baseline visual acuity which showed no improvement on follow-up, suggesting it to be poor prognostic biomarker.

## Introduction

Central retinal artery occlusion (CRAO), is a devastating ophthalmic emergency first described by Von Graefes in the year 1859 [1]. It presents with sudden onset painless gross diminution of vision with classical clinical findings of retinal opacification and a cherry red spot on fundus examination [2–4]. The acute obstruction to retinal blood flow is most commonly due to an embolus from the carotid artery and/or heart [2–4].

With the introduction of optical coherence tomography technology (OCT), our understanding and management of retino-choroidal diseases have improved significantly. The typical OCT findings of CRAO include inner retinal layer hyperreflectivity, thickening and loss of stratification of retinal layers [5, 6]. These changes are directly proportional to the severity of hypoperfusion and ischemic insult and can be categorized into incomplete, sub-total and total sub-types [5]. These changes of the acute phase gradually resolve, resulting in thinned atrophic inner retinal layers.

Presence of internal limiting membrane detachment (ILMD), a vitreo-retinal interface abnormality has been infrequently noted in acute CRAO [5, 7]. Associated with poor prognosis, ILMD has been proposed to result from total retinal artery occlusion with severe retinal ischemia. However, preliminary work is limited by small sample size and lack of corroboration of this structural OCT finding with blood flow patterns on FFA and OCTA at baseline and follow-up. We report the clinical features, multi-modal imaging characteristics and their correlation, and prognostic value of ILMD, a novel OCT biomarker in acute CRAO.

## Methods

This was a retrospective observational study and approval was taken from the Institutional Ethics Committee [LEC-BHR-R-08-22-920]. The study adhered to the tenets of the Declaration of Helsinki. Electronic medical records were searched using the keyword “Central Retinal Artery Occlusion” during the study period of January 2018 to December 2021. A standard consent form for electronic data privacy and consent for the use of data for research purpose was filled by the patients at the time of registration.

Acute CRAO was defined as history of recent onset (less than one week duration) sudden painless gross diminution of vision with presence of retinal opacification, cherry red spot and arterial attenuation. Medical records of consecutive cases shortlisted from ERM keyword search, were screened manually to check for inclusion and exclusion criteria. Key eligibility criteria included patient aged eighteen years and above and, OCT imaging available at baseline. We excluded eyes with history of ocular trauma, other retinal vascular diseases, combined occlusion, associated optic nerve diseases, macular degenerations, retinal dystrophies, purtscher retinopathy and prior vitreo-retinal surgeries.

All patients underwent a detailed ocular evaluation including BCVA testing, dilated fundus examination with slit-lamp bio-microscopy and OCT (Topcon TRITON 3D PLUS Version 10.19; Topcon, Japan). FFA (Carl Zeiss FP 450, Germany) and OCTA (Topcon TRITON 3D PLUS, Version 10.19, Topcon, Japan) data was collected, where available. Data collected and tabulated included age, gender, systemic co-morbidities, baseline BCVA, anterior chamber paracentesis if done, duration of follow-up, and final BCVA. BCVA was assessed using the Snellen`s chart and listed as the logarithm of the minimum angle of resolution (LogMAR) equivalents for statistical analysis [8, 9].

Of the 18 eyes with ILMD on OCT, seven and four eyes had OCTA and FFA at baseline (two eyes had both FFA and OCTA). Due to significant motion artifact and poor image acquisition of OCTA, four eyes were excluded. On OCT, in-built measurement calipers were used to manually calculate the extent of ILMD on horizontal line scan at fovea. Similarly, horizontal extent of enlarged FAZ was calculated using in-built calipers on a 6X6 mm macular OCTA scan, after manual segmentation correction if needed. In FFA, enlarged FAZ was calculated using measurement tool in Visupac system. All measurements were performed independently by two investigators (MJN & BB) and average of two measurements was used.

The statistical analysis was performed using SPSS v20 for windows (SPSS, Inc, Chicago, Illinois, USA). The distribution of continuous data were checked for normality by Shapiro–Wilk test. Summary measures included mean with standard deviation and proportions for continuous and categorical data, respectively. Mann-Whitney U-test and chi-square test were used to compare means and proportions, respectively across the groups. A binary logistic regression was done to find the possible predictors of ILMD. A p-value of < 0.05 was considered statistically significant.

## Results

Overall, a total of 60 eyes of 60 patients with acute CRAO were included in this study. Eighteen (30%) eyes were noted to have ILMD on OCT scans.

### Baseline characteristics of eyes with ILMD

The mean age at presentation was 47.78 ± 14.9 years with fifteen (83.3%) being males. The mean duration of symptoms was 5.5 ± 3.3 days with a mean visual acuity of 2.62 LogMAR units (Light perception) at presentation.

### Qualitative multi-modal imaging corroborations at baseline

**Fig. 1:**
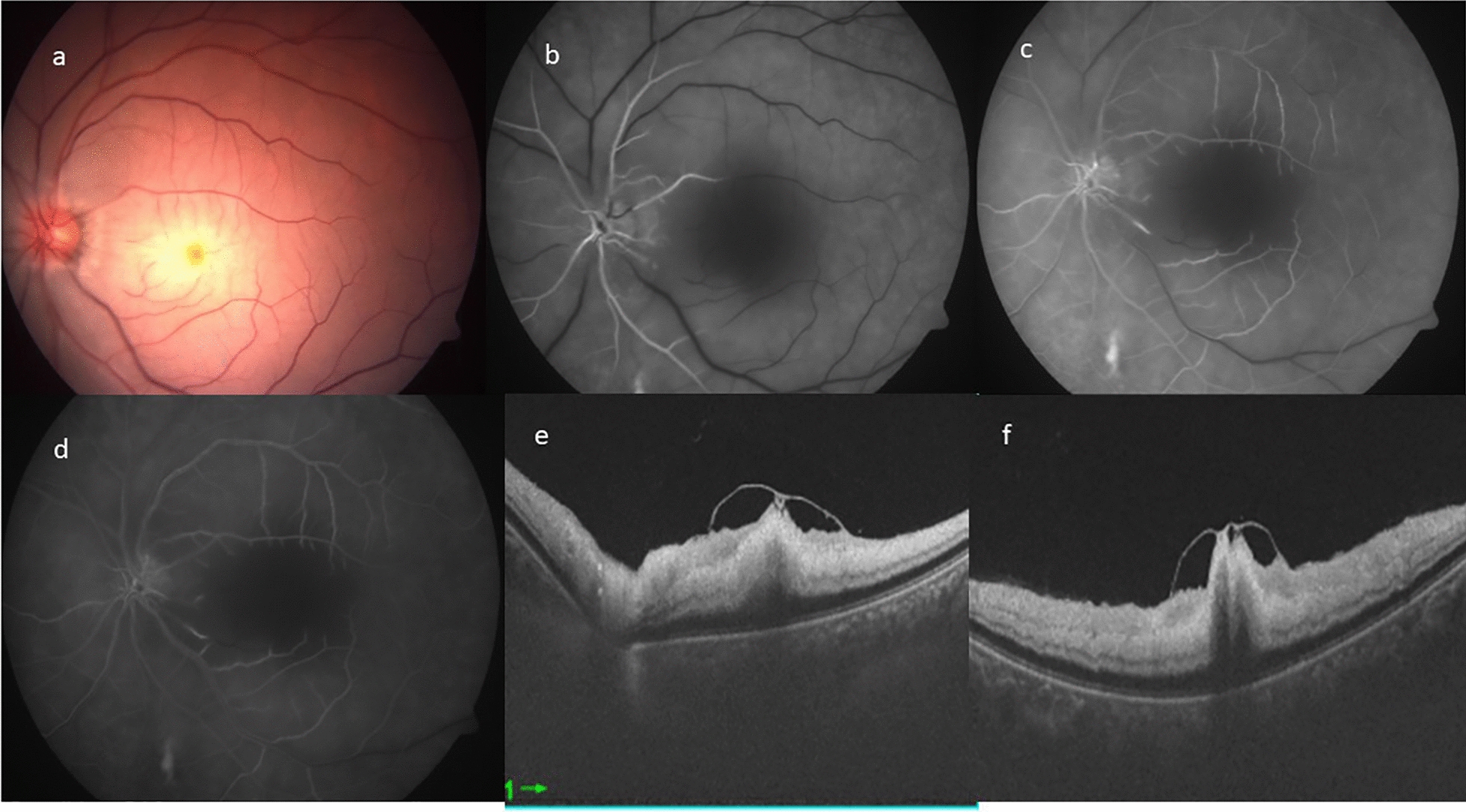
**a** Left eye color fundus photograph of posterior pole, showing widespread retinal opacification with a central cherry red spot at fovea. **b**–**d** Fundus fluorescein angiography. **b** Extremely delayed arterial filling with the leading edge of the dye noted. **c** Marked delay in the arterio-venous transit time, with hardly any dye reaching the capillaries. **d** Late phase showing washout of dye with some laminar filling of veins close to the disc is noted. Significant central capillary filling defect resulting in enlargement of foveal avascular zone is noted. Optical coherence tomography horizontal **e** and vertical **f** scans showing inner retinal layers hyperreflectivity, edema and loss of stratification with a localized internal limiting membrane detachment with central foveal attachment

**Fig. 2:**
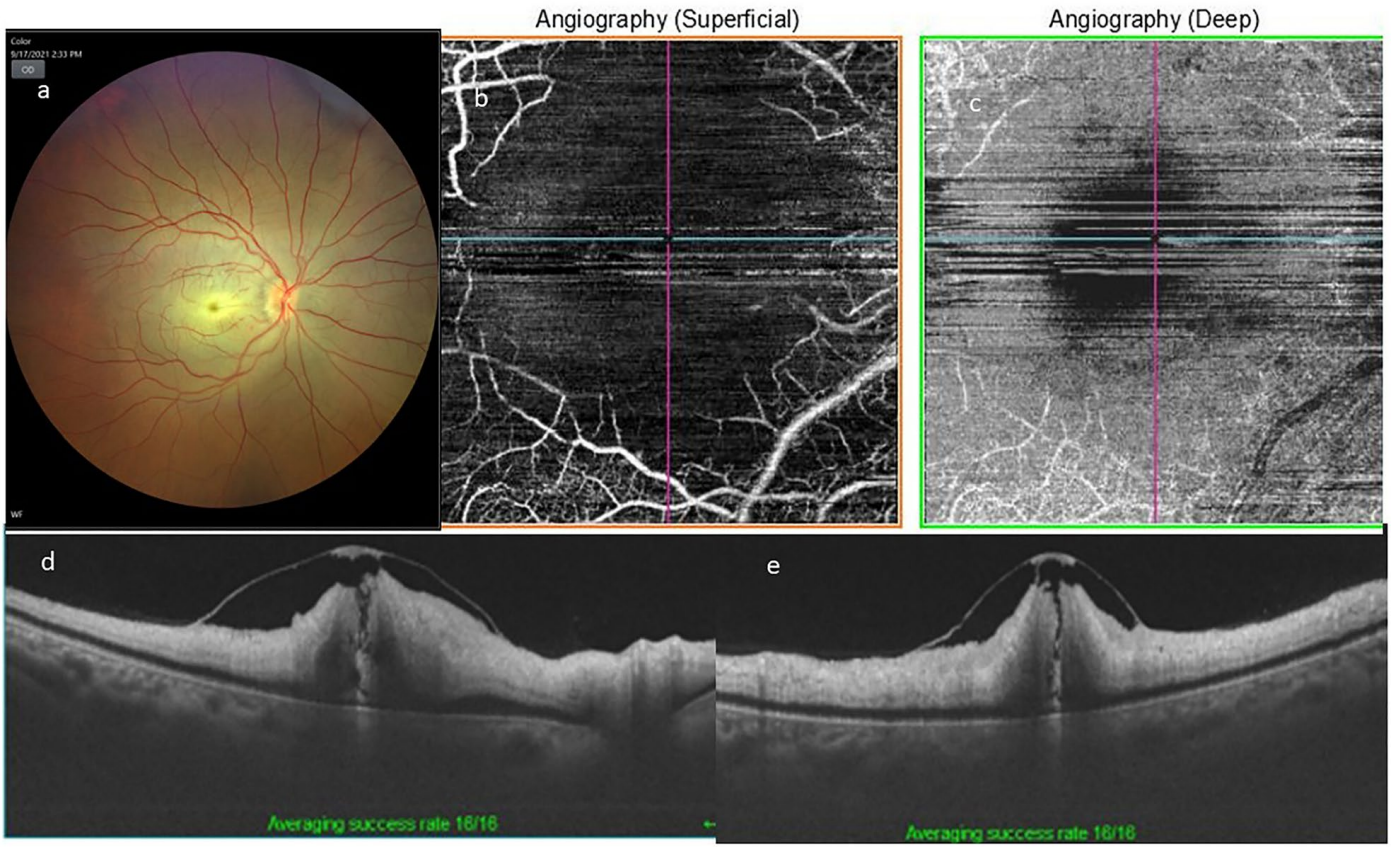
**a** Right eye color fundus photograph of the posterior pole, showing widespread retinal opacification with a central cherry red spot at fovea. **b**, **c** Optical coherence tomography angiography superficial (**b**) and deep (**c**) capillary network. Pruning of both arteries and veins with significant capillary flow void areas in the macular region. Presence of some motion artifact in the central region is evident, possibly due to poor fixation. Optical coherence tomography horizontal (**d**) and vertical (**e**) scans showing inner retinal layers hyperreflectivity, edema and loss of stratification with an localized internal limiting membrane detachment with an overlying “operculum”

**Fig. 3:**
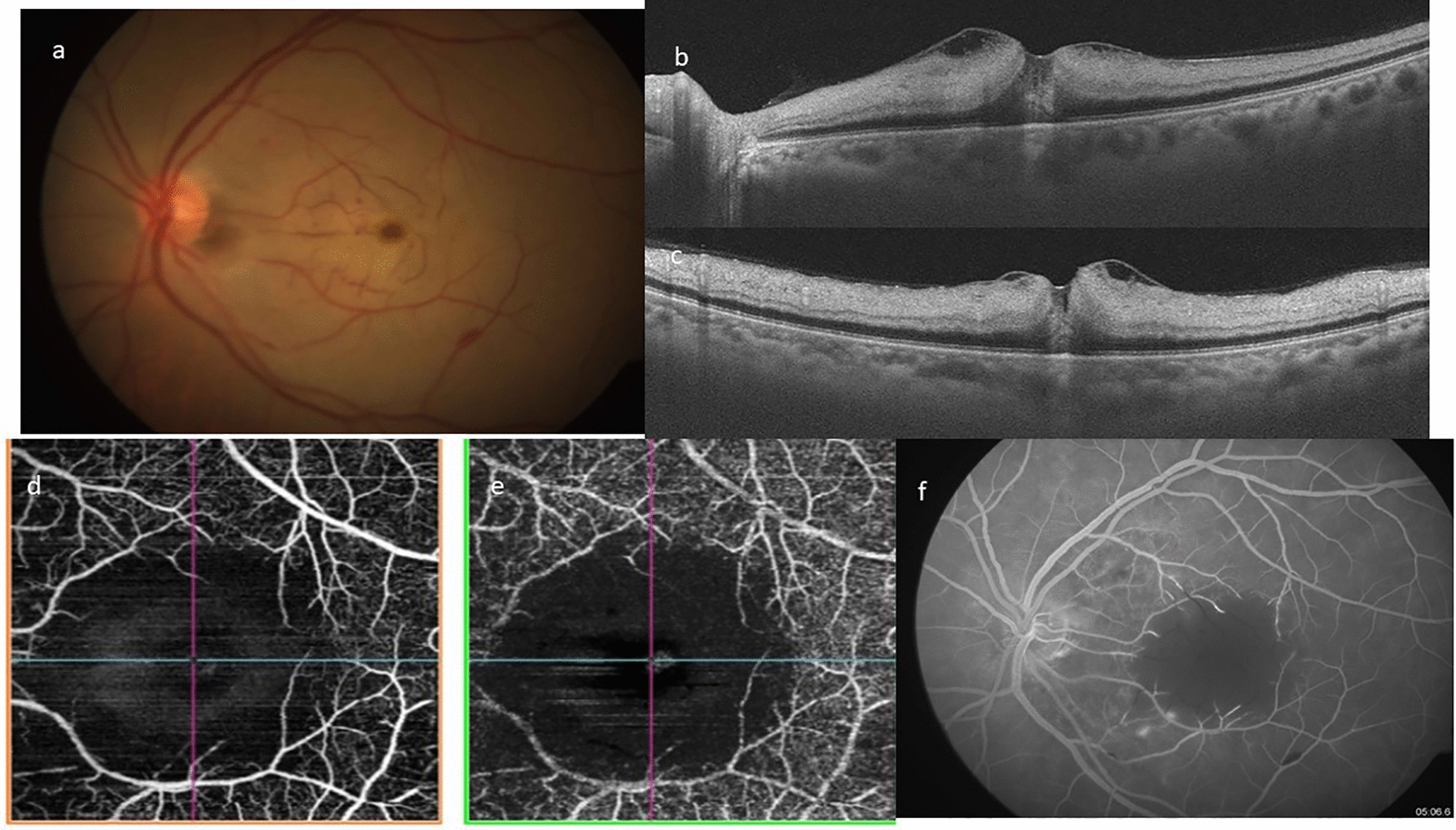
**a** Left eye color fundus photograph of the posterior pole, showing widespread retinal opacification with a central cherry red spot at fovea. There is presence of box-caring and narrowing of arterioles. **b**, **c** Optical coherence tomography horizontal (**b**) and vertical (**c**) scans showing inner retinal layers hyperreflectivity, edema and loss of stratification with an localized shallow internal limiting membrane detachment with central foveal attachment. **d**, **e** Optical coherence tomography angiography superficial (**d**) and deep (**e**) capillary network. Pruning of both arteries and veins with significant capillary bed flow-void areas in the macular region. **f** Fundus fluoresceine angiography of recirculation phase showing washout of dye with significant central capillary filling defect resulting in enlargement of foveal avascular zone

**Fig. 4:**
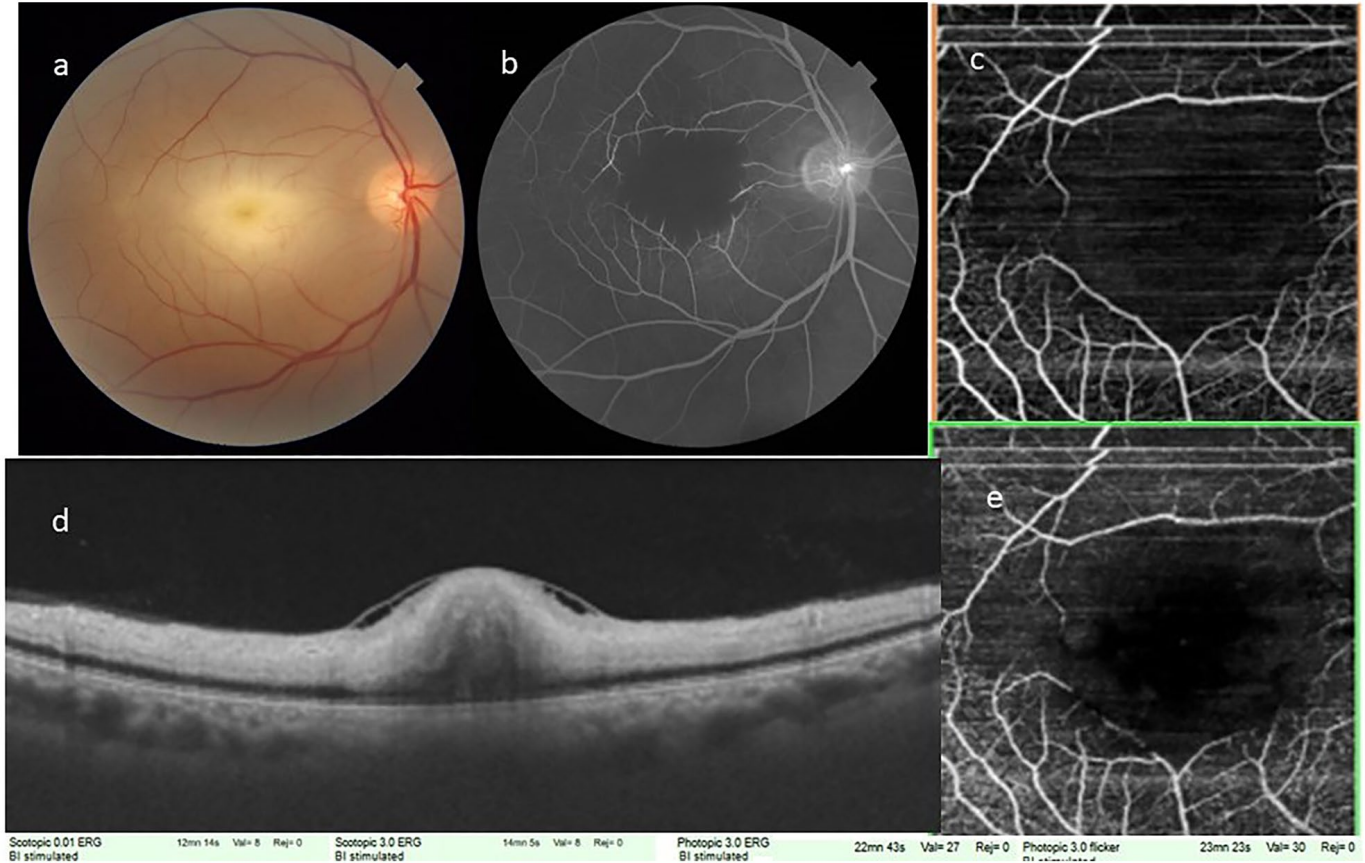
**a** Color fundus photograph posterior pole of the left eye, showing widespread retinal opacification with a central cherry red spot at fovea. **b** Fundus fluoresceine angiography of recirculation phase showing washout of dye with significant central capillary filling defect resulting in enlargement of foveal avascular zone. **c** & **e** Optical coherence tomography angiography superficial (**c**) and deep (**e**) capillary network. Pruning of both arteries and veins with significant capillary bed flow void areas in the macular region. **d** Optical coherence tomography vertical scans showing inner retinal layers hyperreflectivity, edema and loss of stratification with an localized shallow internal limiting membrane detachment with central foveal attachment

Multi-modal imaging characteristics of representative cases with ILMD at presentation have been described in Figs. 1, 2, 3, 4.

In all cases, fundus photograph showed widespread retinal opacification and prominent cherry red spot with arterial box-carting and attenuation. No signs of ILMD were identified on fundus photography.

OCT showed classical features of CRAO characterized by diffuse inner retinal thickening, hyper-reflectivity and loss of stratification of inner retinal layers along with attenuation of outer retinal layers. A localized area of ILMD was noted in the parafoveal region with strong attachment to the central fovea, except in case number 2.

FFA in the acute stage showed extremely delayed arterial and arterio-venous transit time. Significant macular capillary filling defect caused enlargement of foveal avascular zone. OCTA in acute stages showed pruning of both arteries and veins with significant macular flow void areas causing an enlarged FAZ.

### Quantitative multimodal imaging characteristics

The mean extent of ILMD on horizontal scan of OCT was 3.82 ± 0.72 mm, which was comparable to the mean FAZ extent on FFA and OCTA was 3.50 ± 0.39 mm and 3.93 ± 0.84 mm, respectively (p > 0.05).

### Qualitative multi-modal imaging corroborations at follow-up

**Fig. 5:**
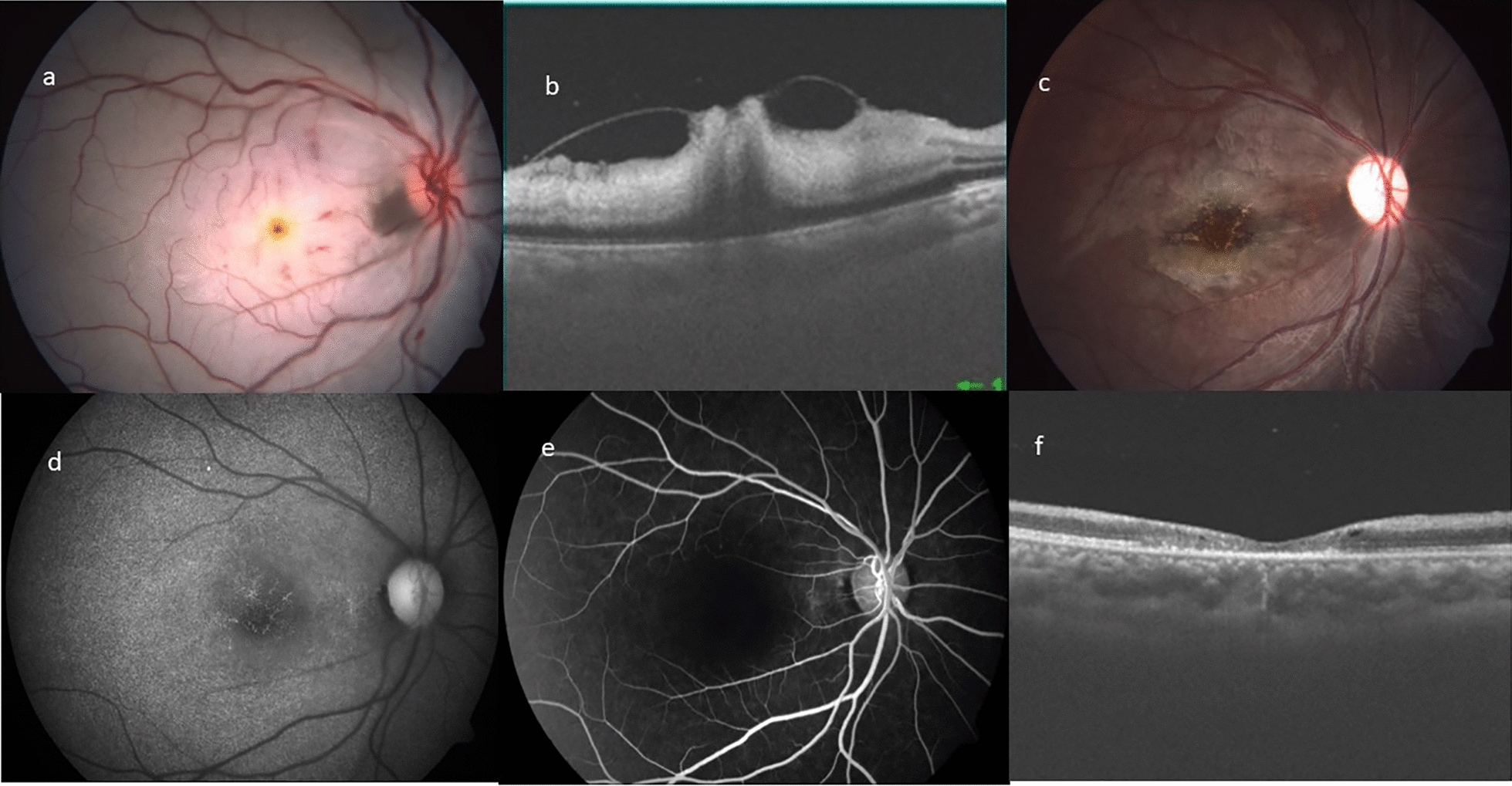
**a** Color fundus photograph posterior pole of the left eye, showing widespread retinal opacification with a central cherry red spot at fovea. **b** Optical coherence tomography vertical scans showing inner retinal layers hyperreflectivity, edema and loss of stratification with an localized shallow internal limiting membrane detachment with central foveal attachment. **c** Color fundus photograph posterior pole of the left eye, showing resolution of retinal opacification with retinal pigment epithelial mottling, most prominent at central macular region. Optic disc appears pale. **d** Fundus autofluorescence showing stippled hypo and hyper-fluorescence at central macular region. **e** Fundus fluoresceine angiography showed improvement in dye transit time, however macula showed “no-flow reperfusion” with an unremitting enlarged foveal avascular zone. **f** OCT scan showing generalized significant thinning of the inner retinal layers and degenerative cystoid changes. At central foveal region, these changes are most marked with disruption of the ellipsoid zone and REP irregularity. ILM is re-attached

**Fig. 6:**
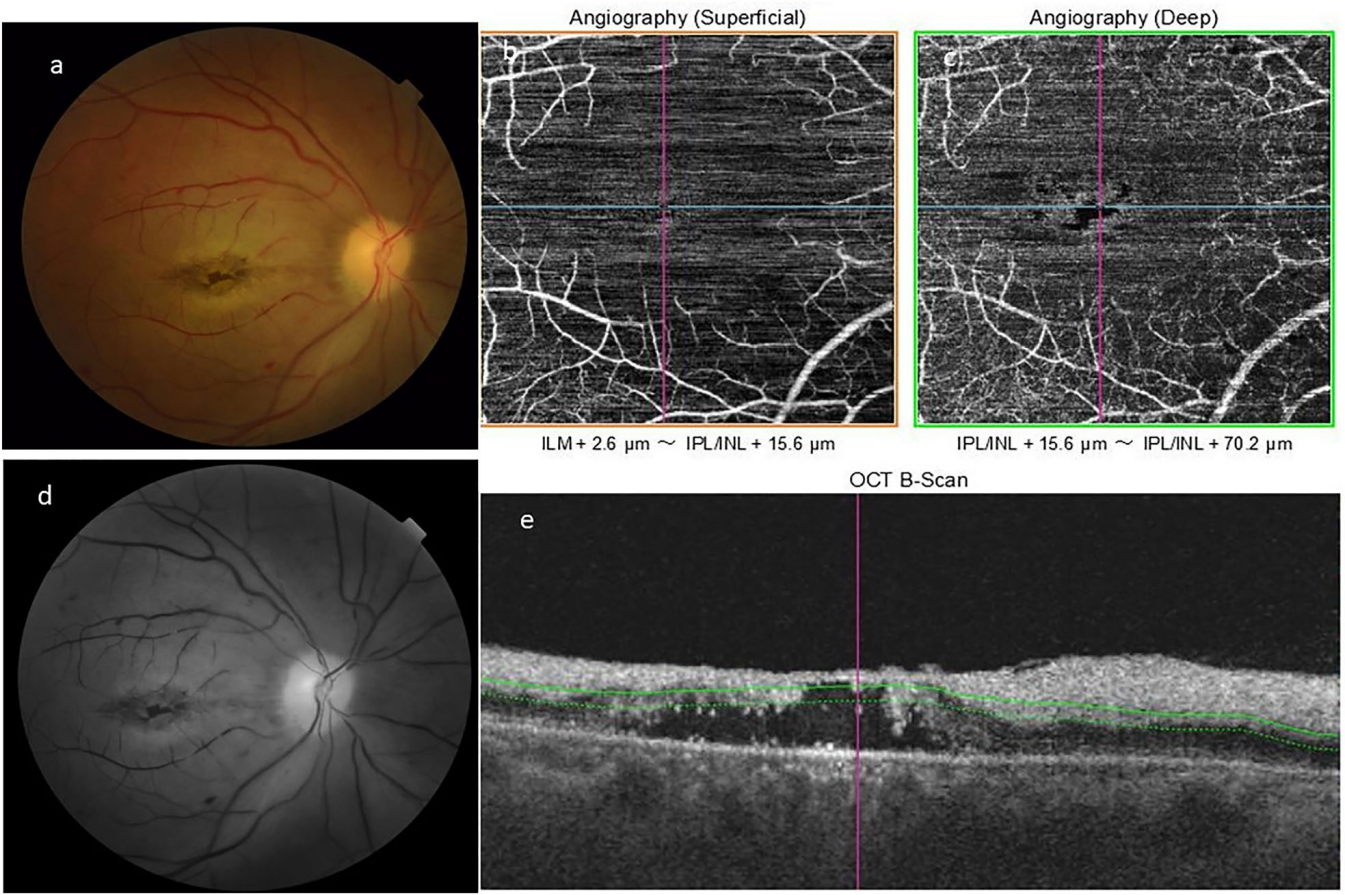
[Follow up case number 2] **a** Left eye color fundus photograph of the posterior pole, showing resolution of retinal opacification except at the para-foveal region with retinal pigment epithelial mottling at fovea. **b**, **c** Optical coherence tomography angiography superficial (**b**) and deep (**c**) capillary network. Pruning of both arteries and veins with persistent capillary bed non-perfusion and enlarged foveal avascular zone. (**d**) Red free photograph posterior pole showing REP changes at fovea with surrounding retinal opacification. (**e**) Optical coherence tomography vertical scans showing reduction in the inner retinal layers hyperreflectivity and edema. At foveal region, there is significant loss of neuro-sensory tissue with complete loss of photo-receptors

Multi-modal imaging characteristics of representative cases with ILMD at presentation have been described in Figs. 5, 6.

On follow-up, fundus photography showed widespread retinal pigment epithelium mottling, most prominent at macular region with disc pallor. OCT on follow up showed resolution of inner layer hyper-reflectivity and edema, with atrophy and thinning. In addition, fragmentation, disruption and cystoid degenerative changes were also noted in some cases. ILMD had resolved in all cases during follow-up.

On FFA, there was near normal establishment of retinal perfusion. However, the macula showed little evidence of re-perfusion. Similar findings were noted on OCTA supporting no macular re-perfusion.

There was no statistically significant difference in the mean age, gender distribution, duration of symptoms, follow-up duration between ILMD and no-ILMD group.Table 1 Mean BCVA at baseline in the group with ILMD as compared to those without ILMD was 2.62 ± 0.29 LogMAR (light perception) versus 2.05 ± 0.52 LogMAR (Snellen equivalent 20/2000, p = 0.000) Table 1.

**Table 1:** Shows the comparison between eyes with ILMD and those without ILMD at presentation

	ILMD (n = 18)	NO ILMD (n = 42)	P
Age	47.78 ± 14.94	48.76 ± 14.91	0.790^@^
Gender (Male/Female)	15/3	28/14	0.228^#^
Systemic disease (YES/NO)	6/12	15/27	0.86^#^
Duration of symptoms	5.5 ± 3.31	4.40 ± 3.65	0.095^@^
Baseline BCVA	2.62 ± 0.29	2.05 ± 0.52	**0.000**^@^
AC paracentesis	6/12	10/32	0.445^&^
Follow up (Days)	48.44 ± 90.82	97.14 ± 153.69	0.155^@^
Final BCVA	2.62 ± 0.29	1.87 ± 0.78	**0.000**^@^

*BCVA* best corrected visual acuity, *AC* anterior chamber, *ILMD* internal limiting membrane detachment

^@^Man-Whitney U test

^#^Fisher Exact test

^&^Chi-square Test

Bold values indicate p<0.05

At the final visit in the ILMD group, BCVA was similar to that at baseline, suggestive of no improvement in any of the 18 eyes. In comparison, in no-ILMD group showed improvement with a final visual acuity of 1.87 ± 0.78 LogMAR (Snellen equivalent 20/1400, p = 0.000). Nine (21.4%) eyes in no-ILMD group had improvement to 20/400 and above, as compared to none in ILMD group, respectively.

The findings of logistic regression Table 2 showed that poor baseline visual acuity was significantly associated with the odds of ILMD (Odds Ratio 31.02, p = 0.0018, 95% confidence interval – 1.81–529) while controlling for potential confounders including age (p = 0.60), gender (p = 0.316) duration of symptoms (p = 0.114), follow-up duration (p = 0.450) and final BCVA (p = 0.357).

**Table 2:** Shows summary of multivariate binary logistic regression analysis

	Odds Ratio	5% C.I	95% C.I	P
Age	1.01	0.961	1.072	0.60
Gender	2.54	0.411	15.67	0.316
Duration of Symptoms	1.12	0.955	1.53	0.114
Baseline BCVA	31.02	1.81	529.10	**0.018**
AC paracentesis	3.26	0.584	18.202	0.178
Follow-up	1.00	0.998	1.005	0.450
Final BCVA	2.09	0.435	10.077	0.357

*BCVA* best corrected visual acuity, *AC* anterior chamber, *ILMD* internal limiting membrane detachment, *CI* confidence interval

Bold values indicate p<0.05

## Discussion

Our study suggests that ILMD is a novel OCT biomarker associated with poor prognosis in acute CRAO. On multi-modal imaging, ILMD correlated with macular non-perfusion, and resulted in pronounced retinal thinning and atrophy on follow-up. Compared to eyes with no ILMD, eyes with ILMD had poor baseline visual acuity equivalent to perception of light and showed no improvement on follow-up, validating its utility as prognostic predictor.

CRAO results from obstruction to the blood flow in the central retinal artery which supplies the inner retina. Obstruction can be due to an emboli, thrombus, inflammation/trauma induced vessels wall damage or spasm. Short duration of ischemia is well tolerated without any detectable functional or anatomical damage [10, 11]. However, when ischemic insult exceeds a critical duration, end organ damage happens [10, 11]. This results in cell death by apoptotic and autophagic mechanism and results in thinning and atrophy [12].

Muller cells span across the thickness of retina and provide major architectural support [13]. Different cells lines react differently to the same degree of ischemic insult. Previous research have shown that neuronal cells are more susceptible to ischemic insult that glial cell [14]. We found all eyes with ILMD had evidence of profound macular ischemia on OCT, FFA and OCTA. We hypothesize that with increasing degree of ischemia, more Muller cell gets injured. This leads to structural disintegration and leaves ILM unsupported, leading to ILMD. Furthermore, significant atrophy even to the extent of severe tissue loss in OCT on follow up lend support to the severe ischemic damage as the underlying mechanism []. Qualitative analysis further showed that the extent of ILMD correlated significantly with the enlarged FAZ.

Follow-up FFA and OCTA showed absence of macular re-perfusion and persistence of enlarged FAZ similar to that at presentation. Eyes with ILMD showed poor baseline visual acuity equivalent to light perception with no improvement in follow-up, validating it to be poor prognostic sign.

There are a few limitations of our study. We had a relatively small sample size of eyes with this novel OCT findings. The retrospective design resulted in less data from OCTA and FFA. Vasculature identification in OCTA depends on cell movement within the capillaries. Present available algorithms do not allow identification of very high or low flow. Though it is possible that the vascular assessment was compromised by a low capillary flow after CRAO, it seems unlikely as OCTA correlated significantly with FFA. Due to poor fixation in CRAO eyes, motion artifacts in some of the OCTA scans were present. However, notwithstanding the short-comings of our study, the results substantially lend support to the use of ILMD as a reliable prognostic marker predicting poor outcome in CRAO.

In conclusion, we conclude that ILMD in acute CRAO occurs due to severe macular ischemia and is associated with poor visual prognosis.

## Data Availability

Data underlying this article are available upon reasonable request. Data cannot be made publicly available for ethical and legal reasons. Public availability may compromise participant privacy. Requests for data should be addressed to Dr Raja Narayanan (narayanan@lvepi.org) who will provide the data access in accordance with the institute ethics board policies.

## Abbreviations

CRAO: Central retinal artery occlusion
LogMAR: Logarithm of the minimum angle of resolution
OCT: Optical coherence tomography
OCTA: Optical coherence tomography angiography
FFA: Fundus fluoresceine angiography
BCVA: Best corrected visual acuity
